# Supplementary material for: Genetic code expansion reveals site-specific lactylation in living cells reshapes protein functions
Source: Nat Commun. 2025 Jan 8;16:227. doi: 10.1038/s41467-024-55165-2 (PMC11711764; doi:10.1038/s41467-024-55165-2)
Supplement: Supplementary file 1 — Supplementary Information [file 41467_2024_55165_MOESM1_ESM.pdf]

Supplementary Information

Genetic code expansion reveals site-specific lactylation in living cells reshapes protein functions

Chang Shao[1,†], Shuo Tang[2,†], Siqin Yu[1,†], Chenguang Liu[1], Yueyang Zhang[1], Tianyan Wan[2], Zimeng He[1], Qi Yuan[1], Shihan Wu[2], Hanqing Zhang[1], Ning Wan[1], Mengru Zhan[2], Hui Ye[1,*], Nanxi Wang[2,*]

[1] Jiangsu Provincial Key Laboratory of Drug Metabolism and Pharmacokinetics, State Key Laboratory of Natural Medicines, China Pharmaceutical University, Tongjiaxiang No. 24, Nanjing 210009, Jiangsu, China.
[2] School of Pharmacy, Nanjing University of Chinese Medicine, Xianlindadao No. 138, Nanjing 210023, Jiangsu, China.

[†] These authors contributed equally to this manuscript.

* Correspondence: cpuyehui@cpu.edu.cn (H.Y.) nanxi.wang@njucm.edu.cn (N.W.)

Supplementary Figures

a **Summary of ALDOA-K147 Occupancy**

Cell line	K562-R1	K562-R2	Jurkat-R2	A549-R2	Lung fibro.-R1	HL60-R2	Colon sph.-R1	Colon sph.-R2	HaCat-R1	HAOEC-R1	HAOEC-R2	HEK293T-R2
**Occupancy (%)**	4.78	3.03	9.11	50.33	33.26	13.93	6.84	13.18	0.1	0.8	0.22	0.17

b

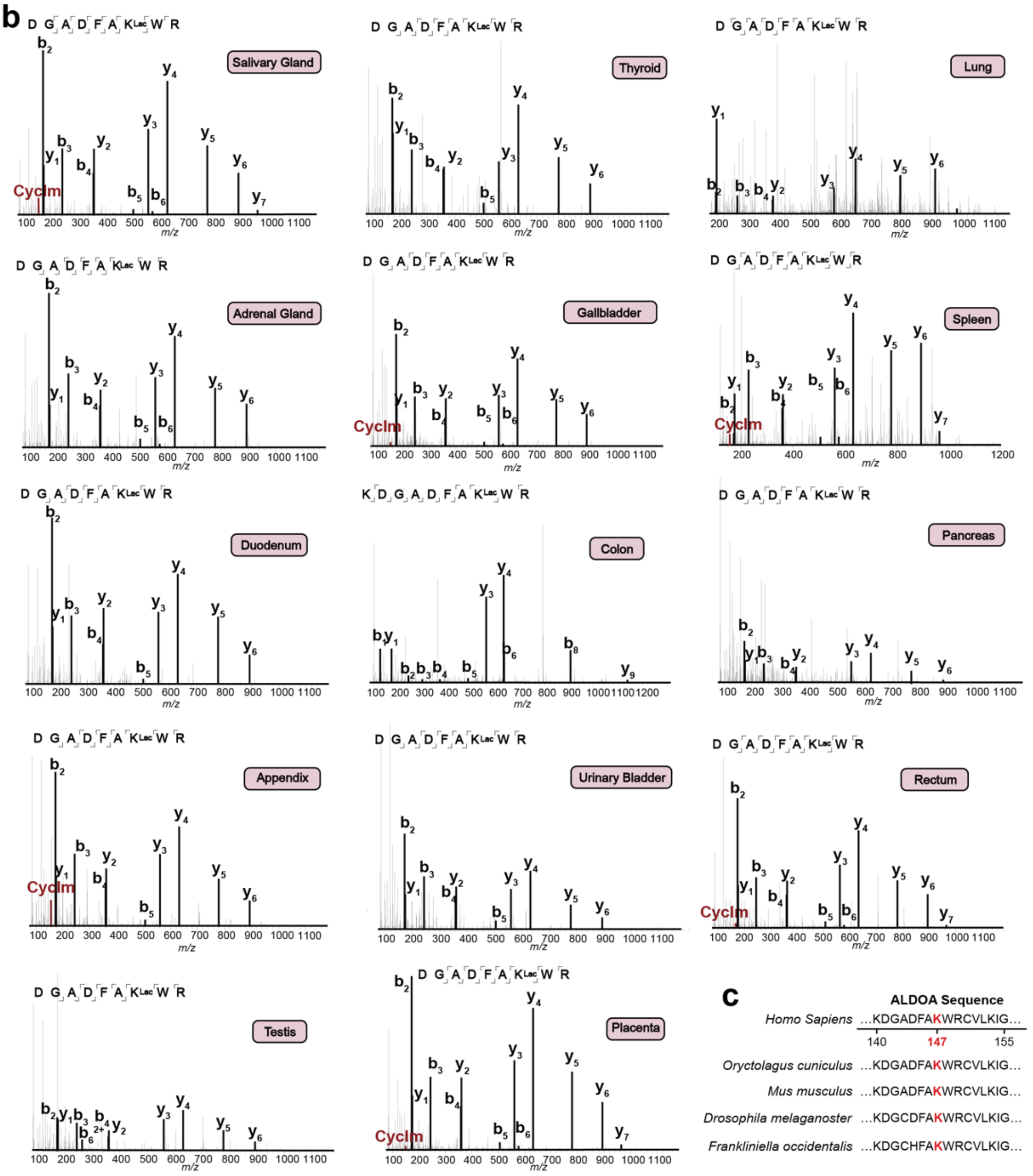

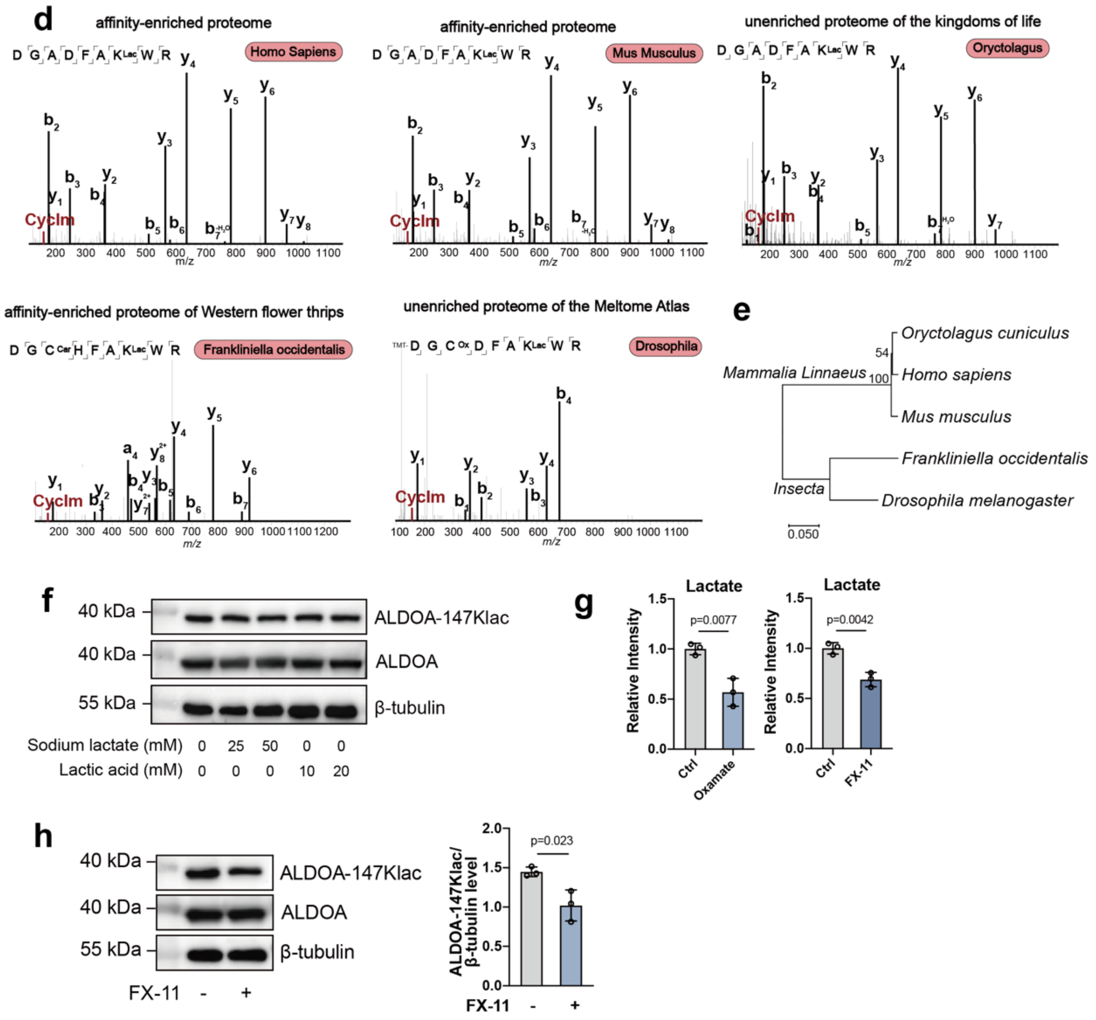

Supplementary Figure 1. Proteomics mining pinpoints functionally important lactylation on ALDOA.

(a) Re-presentation of previously estimated lactylation occupancy for K147 of ALDOA[1] using human cell proteome data retrieved from the Meltome Altas (PXD011929).

(b) Representative MS/MS spectra leading to the assignment of lactylation at K147 of ALDOA in various human tissues, retrieved from the deep proteome atlas of 29 human healthy tissues (PXD010154), including salivary gland, thyroid, lung, adrenal gland, gallbladder, spleen, duodenum, colon, pancreas, appendix, urinary bladder, rectum, testis and placenta.

(c) Evolutionary conservation of K147 on ALDOA across eukaryotic phylogeny.

(d) Representative MS/MS spectra leading to the assignment of lactylation at K147 of ALDOA across 5 species, including *Homo sapiens* (HEK293T) and *Mus musculus* (RAW264.7) from our affinity-enriched lactylproteome, *Oryctolagus cuniculus* from the re-analyzed unenriched proteome of the kingdoms of life (PXD014877), *Drosophila* from the re-analyzed proteome retrieved from the Meltome Altas (PXD011929) and *Frankliniella occidentalis* from an affinity-enriched lactylproteome of Western flower thrips (PXD030799).

(e) Phylogenetic tree representing amino acid sequences of ALDOA from the 5 analyzed species. The neighbor-joining method was used to generate the phylogenetic tree. Bootstrap values were calculated based on 1000 replications. Scale bar indicates the number of amino acid substitutions per site.

(f) Immunoblotting analysis of HEK293T cells treated with the indicated concentrations of sodium lactate or lactic

acid.

(g) Relative intensity of lactate in HEK293T cells treated with oxamate (25 mM, 24 h) or FX-11 (10 μM, 24 h). Data represent the mean ± S.D. (n=3 biological replicates/group) and the p value was calculated by unpaired two-tailed Student's t-test.

(h) Immunoblots showing ALDOA-147Klac levels in HEK293T cells treated with FX-11 (10 μM, 24 h). Data represent the mean ± S.D. (n=3 biological independent samples) and the p value was calculated by unpaired two-tailed Student's t-test.

Source data are provided as a Source Data file.

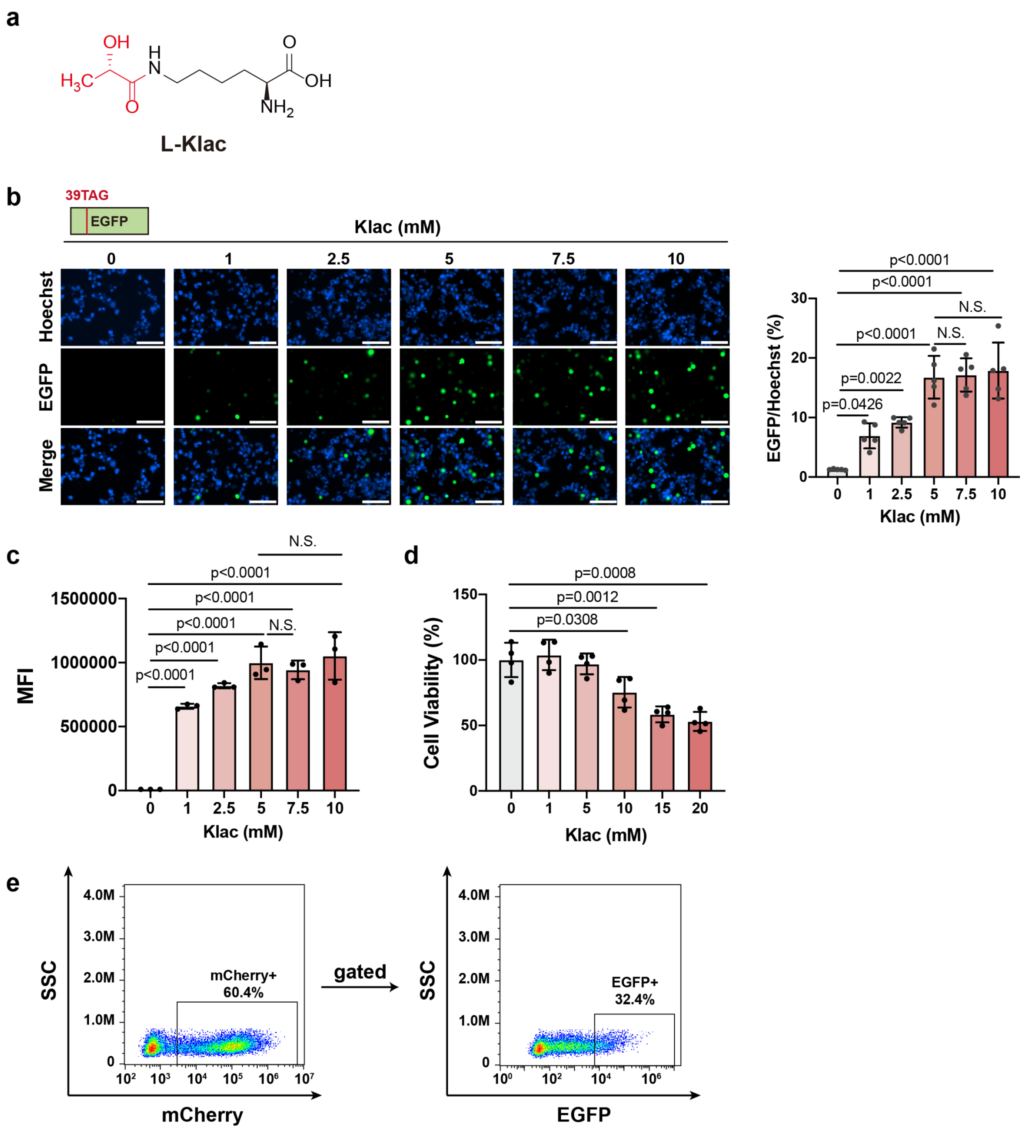

Supplementary Figure 2. Site-specific incorporation of lactylation in mammalian cells with genetic code expansion

(a) Structure of L-Klac.

(b) Representative images of HEK293T cells co-transfected with the KlacRS1/tRNA$^{Pyl}_{CUA}$ pair and EGFP-39TAG plasmids in the presence of the indicated concentrations of Klac (48 h). The nucleus was stained with Hoechst (blue). Scale bar, 100 μm. Data represent the mean ± S.D. (n=5 biological replicates/group) and the p value was calculated by one-way ANOVA.

(c) Flow cytometry analysis of HEK293T cells co-transfected with the KlacRS1/tRNA$^{Pyl}_{CUA}$ pair and EGFP-39TAG plasmids in the presence of the indicated concentrations of Klac (48 h). Data represent the mean ± S.D. (n=3 biological replicates/group) and the p value was calculated by one-way ANOVA.

(d) Cell viability of HEK293T cells treated with the indicated concentrations of Klac (48 h). Data represent the mean ± S.D. (n=4 biological replicates/group) and the p value was calculated by one-way ANOVA.

(e) Comparison of Klac incorporation efficiency in HEK293T cells co-transfected with the KlacRS1/tRNA$^{Pyl}_{CUA}$ pair (**Fig 2c**) or chKlacRS-IPYE/tRNA$^{Pyl}_{CUA}$ pair in the presence of Klac (1 mM, 48 h) using mCherry-TAG-EGFP as the reporter.

Source data are provided as a Source Data file.

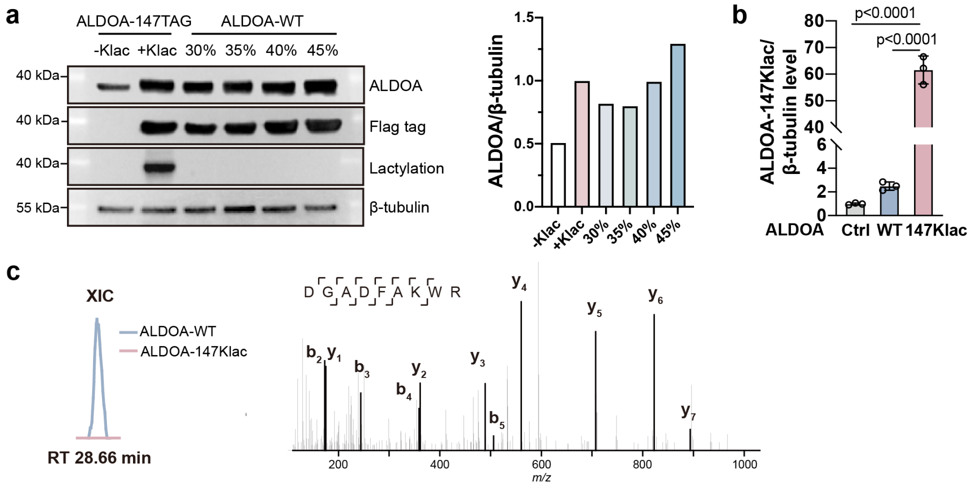

Supplementary Figure 3. Introducing site-specific lactylation at K147 of ALDOA in mammalian cells.

(a) Immunoblotting analysis of HEK293T cells co-transfected with the KlacRS1/tRNA$^{Pyl}_{CUA}$ pair and ALDOA-147TAG plasmids or different amounts of ALDOA-WT plasmids in the presence or absence of Klac (5 mM, 48 h). The expression levels of ALDOA-147Klac and ALDOA-WT were adjusted to comparable (Lane 2 and 5) for subsequent functional studies. Left, representative images of western blots. Right, quantification of ALDOA levels, normalized to ß-tubulin.

(b) Quantification of ALDOA-147Klac levels normalized to ß-tubulin in **Fig. 2e**. Data represent the mean ± S.D. (n=3 biological independent samples) and the p value was calculated by one-way ANOVA.

(c) Identification of non-lactylated ALDOA-K147 peptides by IP-MS analysis. HEK293T cells overexpressing Flag-tagged ALDOA-WT or ALDOA-147Klac, followed by immunoprecipitation with anti-Flag antibody and bottom-up proteomic analysis. Left, XIC of non-lactylated K147-bearing peptide. Right, representative MS/MS spectrum of non-lactylated K147 peptide (with missed cleavage).

Source data are provided as a Source Data file.

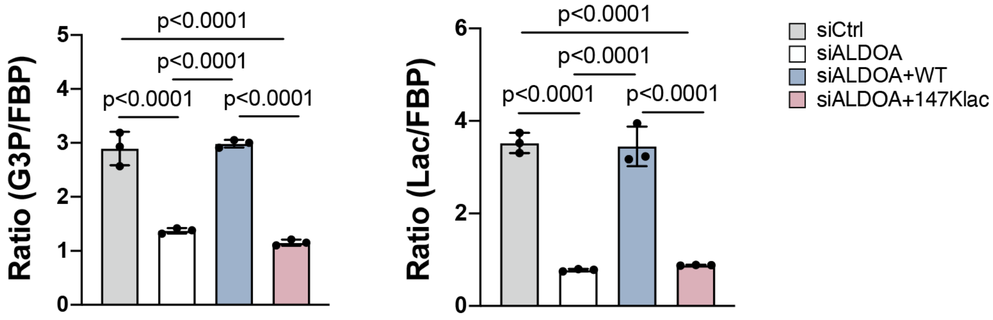

Supplementary Figure 4. Lactylation at ALDOA-K147 abolished enzyme activity and impaired glycolytic flux. Abundance ratios of G3P/FBP and Lac/FBP in the siCtrl, siALDOA, siALDOA+WT and siALDOA+147Klac groups. Data represent the mean ± S.D. (n=3 biological replicates/group) and the p value was calculated by one-way ANOVA.

Source data are provided as a Source Data file.

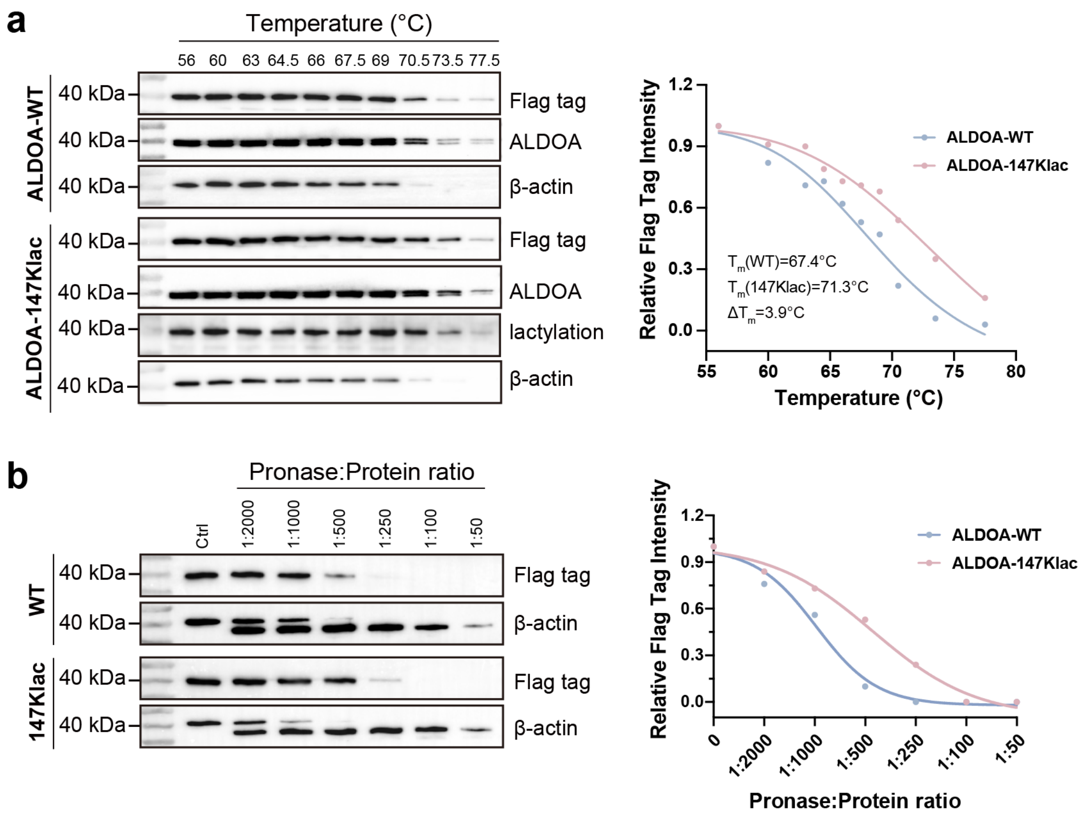

Supplementary Figure 5. Site-specific lactylation modulated ALDOA stability.

(a) Immunoblotting-based CETSA using HEK293T cells overexpressing either Flag-tagged ALDOA-WT or ALDOA-147Klac. Left, representative images of western blots. Right, band intensity profiles. Melting temperature (T_m) is defined as the temperature at which a reduction of 50% signal (soluble protein) is observed. Experiments were repeated three times (n=3 biologically independent samples) yielding consistent results, and one set of representative data was presented.

(b) Immunoblotting-based DARTS assay using HEK293T cells in **(a)**. Left, representative images of western blots. Right, band intensity profiles. Experiments were repeated three times (n=3 biologically independent samples) yielding consistent results, and one set of representative data was presented.

Source data are provided as a Source Data file.

a

Location	Probability of Location
Cytoplasm	90.6%
Nucleus	9.2%
Mitochondrion	0.2%

b

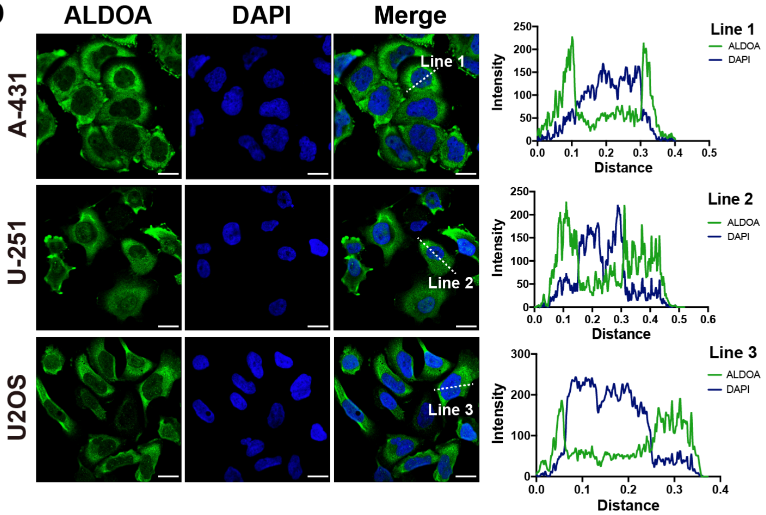

Supplementary Figure 6. Subcellular localization analysis of ALDOA-WT indicated its primary localization in the cytoplasm.

(a) Subcellular localization of ALDOA was predicted via YLoc[2] analysis with a confidence score of 0.82 (https://abi-services.cs.uni-tuebingen.de/yloc/webloc.cgi).

(b) Subcellular localization of unmodified ALDOA in human cell lines A-431, U-251 and U2OS by immunofluorescence staining analysis retrieved from the Human Protein Altas[3] (https://www.proteinatlas.org/ENSG00000149925-ALDOA/subcellular). Cells were stained with ALDOA (green) and the nucleus was labeled with DAPI (blue). Left, representative images showing that ALDOA was primarily localized in the cytoplasm. Right, fluorescence intensity profiles across the lines indicated on the left. Scale bar, 20 μm.

Source data are provided as a Source Data file.

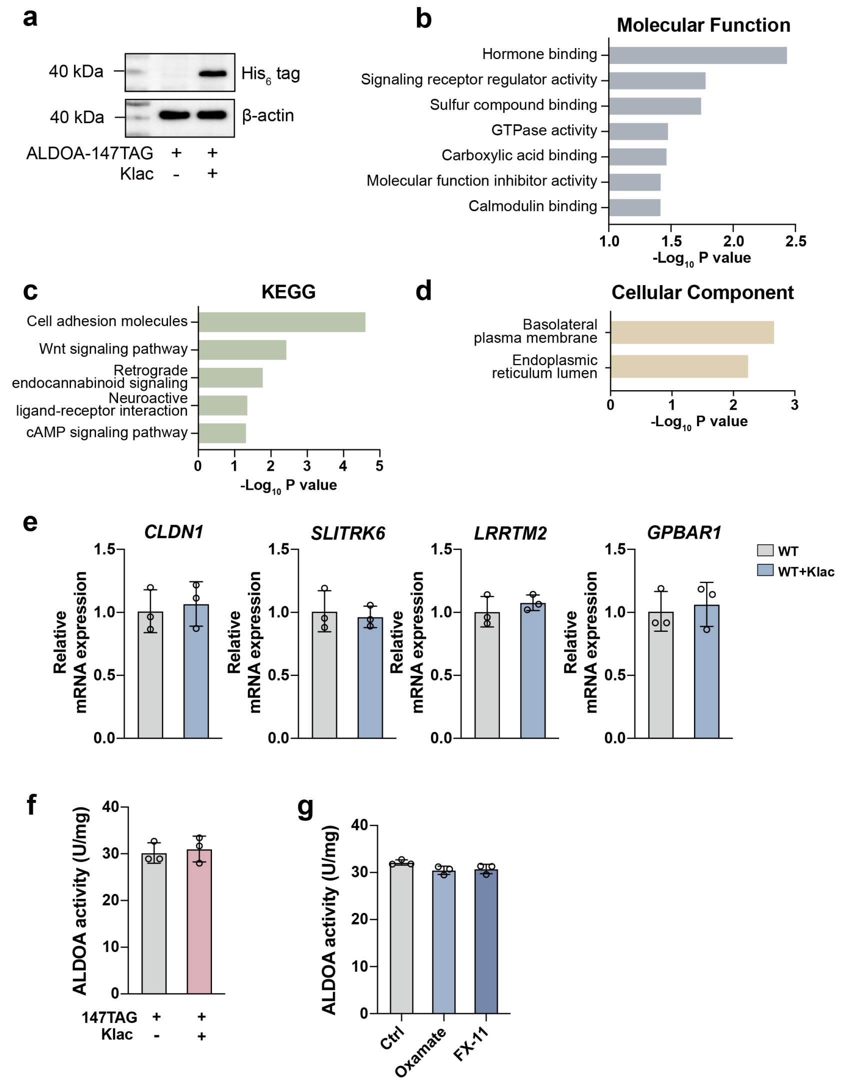

Supplementary Figure 7. Lactylation on ALDOA induced transcriptional changes in living cells.

(a) HEK293T cells co-transfected with the KlacRS1/$tRNA^{Pyl}_{CUA}$ pair and ALDOA-147TAG plasmids in the presence or absence of Klac (5 mM, 48 h) were used for RNA-seq analysis.

(b-d) Bar chart of the GO MF analysis (b), CC analysis (c) and the KEGG pathway analysis (d) for the differentially regulated genes using Metascape. The p values were calculated using one-sided Fisher's exact test and adjusted by the Benjamini-Hochberg method.

(e) RT-qPCR analysis of *CLDN1*, *SLITRK6*, *LRRTM2* and *GPBAR1* expression in cells transfected with ALDOA-WT with and without Klac (5 mM, 48 h). The endogenous β-tubulin gene was used as the internal control for normalizing target gene expression changes. Data represent the mean ± S.D. (n=3 biological replicates/group) and the p value was calculated by one-way ANOVA.

(f) ALDOA activity of HEK293T cells co-transfected with the KlacRS1/$tRNA^{Pyl}_{CUA}$ pair and ALDOA-147TAG plasmids in the presence or absence of Klac (5 mM, 48 h). Data represent the mean ± S.D. (n=3 biological replicates/group)

(g) ALDOA activity of HEK293T cells treated with or without oxamate (25 mM, 24 h) and FX-11 (10 µM, 24 h). Data represent the mean ± S.D. (n=3 biological replicates/group)

Source data are provided as a Source Data file.

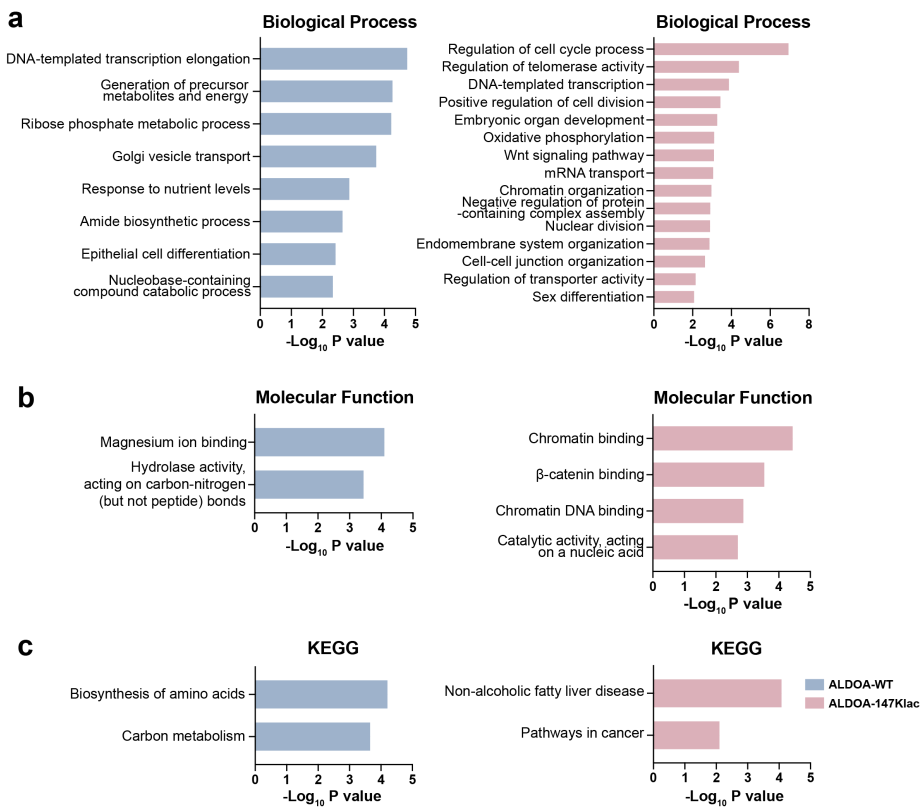

Supplementary Figure 8. ALDOA recruited distinct interacting partners after lactylation.

(a-c) Bar chart of the GO BP analysis (a), MF analysis (b), and the KEGG pathway analysis (c) of the interacting proteins enriched for Flag-tagged ALDOA-WT and ALDOA-147Klac performed by Metascape. The p values were calculated using one-sided Fisher's exact test and adjusted by the Benjamini-Hochberg method.

Source data are provided as a Source Data file.

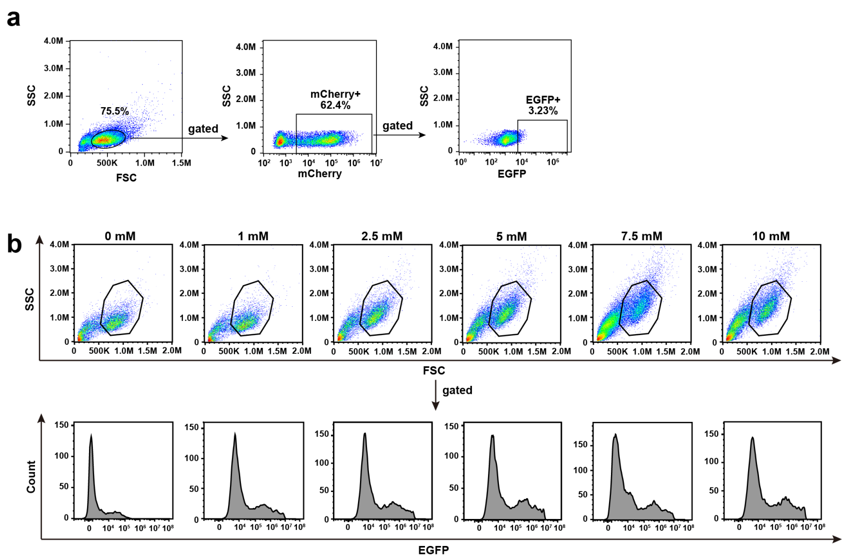

Supplementary Figure 9. Flow cytometry gating strategy.

(a) Flow cytometry gating strategy for Fig. 2c and Fig. S2e. (b) Flow cytometry gating strategy for Fig. S2c.

References

1. Wan, N. *et al.* Cyclic immonium ion of lactyllysine reveals widespread lactylation in the human proteome. *Nat Methods* **19**, 854-864 (2022).
2. Briesemeister, S., Rahnenfuhrer, J. & Kohlbacher, O. YLoc--an interpretable web server for predicting subcellular localization. *Nucleic Acids Res* **38**, W497-502 (2010).
3. Thul, P.J. *et al.* A subcellular map of the human proteome. *Science* **356** (2017).
