# Supplementary material for: Genetic code expansion reveals site-specific lactylation in living cells reshapes protein functions
Source: Nat Commun. 2025 Jan 8;16:227. doi: 10.1038/s41467-024-55165-2 (PMC11711764; doi:10.1038/s41467-024-55165-2)
Supplement: Supplementary file 3 — Description of Additional Supplementary Files [file 41467_2024_55165_MOESM3_ESM.pdf]

Description of Additional Supplementary Files

Supplementary Data 1. Summary of the lactylated peptides identified from the 12 cell lines.

Supplementary Data 2. Summary of the peptides carrying ALDOA-147Klac identified from 15 healthy tissues retrieved from the deep proteome atlas of 29 healthy human tissues.

Supplementary Data 3. Occupancy of ALDOA-147Klac quantified from 15 healthy tissues retrieved from the deep proteome atlas of 29 healthy human tissues.

Supplementary Data 4. Summary of the peptides carrying ALDOA-147Klac identified from different organisms.

Supplementary Data 5. DNA sequences of KlacRS variants used in this study.

Supplementary Data 6. Sequences of PCR Primers used in this study.

Supplementary Data 7. Summary of the interacting proteins for ALDOA-WT and ALDOA-147Klac identified from HEK293T cells.
